# Stimulating Employability and Job Crafting Behaviour of Physicians: A Randomized Controlled Trial

**DOI:** 10.3390/ijerph19095666

**Published:** 2022-05-06

**Authors:** Evelien H. van Leeuwen, Eva Knies, Elizabeth L. J. van Rensen, Toon W. Taris

**Affiliations:** 1 Department of Quality and Patient Safety, University Medical Center Utrecht, Heidelberglaan 100, 3584 CX Utrecht, The Netherlands; e.l.j.vanrensen@umcutrecht.nl; 2 School of Governance, Utrecht University, Bijlhouwerstraat 6, 3511 ZC Utrecht, The Netherlands; e.knies@uu.nl; 3 Department of Social, Health and Organizational Psychology, Utrecht University, Heidelberglaan 1, 3584 CS Utrecht, The Netherlands; a.w.taris@uu.nl

**Keywords:** work ability, willingness to work, employability, job crafting, physicians, intervention study, field experiment

## Abstract

The demanding work context of physicians challenges their employability (i.e., their ability and willingness to continue to work). This requires them to proactively manage their working life and employability, for instance, through job crafting behaviour. This randomized controlled intervention study aimed to examine the effects of a personalized feedback report on physicians’ employability and job crafting behaviour. A total of 165 physicians from two hospitals in a large Dutch city were randomly assigned to a waitlist control or intervention group in May 2019. Physicians in the intervention group received access to a personalized feedback report with their employability scores, suggestions to improve these and to engage in job crafting. Participants completed a pre-test and eight weeks later a post-test. RM MANOVAs and RM ANOVAs showed that the intervention enhanced participants’ perceptions of their mental (*F* (1,130) = 4.57, *p* < 0.05) and physical (*F* (1,135) = 16.05, *p* < 0.001) ability to continue working. There was no effect on their willingness to continue to work. Furthermore, while job crafting behaviour significantly increased over time, the personalized feedback report did not account for this change. This low-investment intervention is relevant for organizations to stimulate employees’ proactivity and create positive perceptions of their ability to continue to work. Moreover, this study contributes to the literature by examining a novel approach of a job crafting intervention that does not require many resources to implement.

## 1. Introduction

Today’s work environment and jobs are highly dynamic [1,2], which may result in knowledge and skills becoming obsolete fast. The work environment is also described as demanding [2,3], since working lives are extended due to an increase in retirement age and an ageing workforce, e.g., [4]. This may result in increasing job demands that tend to reduce one’s physical and mental well-being [5], thus challenging employees’ employability, defined as their physical and mental ability as well as their willingness to continue to work [6,7]. This is an important notion as employable individuals can better cope with the nature of today’s labour market [8], p. 169, and perform better [9].

This dynamic context requires employees to proactively take control over their working life by creating a resourceful, healthy and motivating environment [10]. Job crafting refers to such proactive employee behaviours [11], which are defined as the “self-initiated behaviours that employees take to shape, mold, and change their jobs” [12], p. 126. Engaging in job crafting can help employees to cope with ongoing changes [13]. Moreover, job-crafting behaviour promotes outcomes such as work engagement [14], performance [15], well-being [3], and job satisfaction [16]. 

The beneficial outcomes of employability [8,17] and job crafting behaviours [14,15] emphasize the need to examine how these can be enhanced. Unfortunately, the insights obtained in previous research are often limited. First, much of the literature on employability has focused on the correlates, antecedents and outcomes of these concepts. However, interventions that can enhance employees’ employability have rarely been examined, meaning that evidence on their effectiveness is limited [18,19].

Second, so far, job-crafting studies have mainly focused on the effects of active training sessions on job crafting behaviour [20,21,22], where a group of participants works intensively on job-crafting exercises for at least half a day. Many of these active training interventions show positive effects. However, these training interventions are costly and time-consuming. It remains unclear whether job crafting behaviour can also be increased in a less intense and time-consuming way.

The present study was designed to fill these gaps. It examines the effects of providing physicians with a personalized feedback report containing self-reported scores on their employability, as well as suggestions for possible ways to improve these and to engage in job-crafting behaviour. That is, we examine a low-investment intervention, referring to a reasonably short-term intervention characterized by self-guidance via technology without involvement of a coach or trainer. This randomized controlled intervention study aims to examine the effects of such a low-investment intervention (specifically, access to a personalized feedback report) on physicians’ employability and job-crafting behaviour. Drawing on data from physicians from two Dutch hospitals in a large city, the central question is: what are the effects of providing a personalized feedback report to physicians on their perceptions of employability (i.e., the ability and willingness to continue working until retirement age) and job-crafting behaviours? This question was analysed using two-way repeated-measures multivariate analysis of variance (RM MANOVA) and repeated-measures ANOVAs (RM ANOVA).

Examining this for physicians is highly relevant as they fulfil an important role in society, as their employability is of vital importance to the healthcare that they provide. Additionally, their work environment is subject to significant changes [23] and high demands [24], which is reflected in high burnout rates among physicians worldwide [25]. This makes attention for finding ways to cope with ongoing changes important for physicians.

The following describes the theoretical background of this study, where the central concepts of this study (employability, job crafting and feedback) are elaborated upon. This is followed by a description of the research methods. Then, the results are presented, focussing on the effects of the intervention on employability and on job crafting. This study ends with a discussion and conclusion, including its implications and suggestions for future research.

### 1.1. Theoretical Background

This study examines the effects of providing a personalized feedback report on physicians’ employability and job crafting behaviour. Including these outcomes is helpful to understand both short-term perceptions of behaviour (job crafting), as well as perceptions of attitudes in the long term (employability). Below, we first elaborate on these concepts. Subsequently, we discuss the relationship between the intervention and these outcomes.

**Employability.** Employability can be studied from various different perspectives, meaning that employability has been conceptualized in various ways [26,27]. In this study, we focus on employees’ (physical and mental) ability as well as their willingness to continue to work in their profession [6,7]. This perspective of employability fits with the ‘personal strengths’ perspective of employability [26], referring to employees’ personal strengths (e.g., their competences or abilities, cf. [28,29]) and attitudes (e.g., their willingness to change or to continue to work [30]) that together promote an individual’s chance of employment. 

Physicians usually have permanent contracts and work in the same profession until retirement [31]. This is due to their long education, high level of specialization [32] and strong professional role identity [33]. Physicians in the Netherlands are either employed by the hospital, which is the case in academic hospitals, or work as self-employed in independent establishments, running partnerships within hospitals. Physicians with both types of employment contracts are unlikely to leave their occupation. This makes it relevant to focus on continuing employment in their current profession. The input-based approach towards employability [34] is in line with this view as it focuses on professionals’ personal strengths that can help them to continue working within their profession.

**Job crafting.** There are two dominant perspectives on job crafting [12,35]. One is based on the original job crafting theory [11] and the other is based on the job demand-resource perspective [36]. Wrzesniewski and Dutton [11] understand job crafting as the physical and cognitive changes individuals make in the task or relational boundaries of their work. Conversely, job crafting from a job demand-resource perspective makes a distinction between two types of job characteristics. First, job demands require sustained psychological and/or physical effort or skills [36]. Hindering job demands are health-impairing and impede optimal functioning [31]. Secondly, job resources are job aspects that refer to those aspects of a job that help to achieve certain goals and that stimulate learning and development [36]. Wrzesniewski et al. [37] reviewed the conceptualizations of job crafting used in earlier research. They argued that most studies into job crafting lacked the element of “creating meaningful work by aligning the job with values, motivations, and beliefs” (p. 289), an element which they introduced in their seminal conceptualization of job crafting in 2001. A new perspective on job crafting, developed by Kooij et al. [22], integrated this idea of meaningful work in the job crafting concept, conceptualized as personal resources. Their scale includes items on job crafting towards personal strengths, referring to the self-initiated changes that individuals make in the task boundaries of their work to use their strengths better. They also included job crafting towards personal interests, meaning “to actively look for tasks that match one’s interests” [22], p. 972. The present study combines both perspectives, since these focus on complementary aspects of job crafting: job crafting towards personal resources [22,37], and job crafting in order to change job characteristics [38].

#### The Role of Feedback

Participants in the intervention group will receive feedback on their employability scores and will receive suggestions for job crafting behaviour. This personalized feedback is expected to result in more positive perceptions of employability and job-crafting behaviour. Many feedback intervention studies have been conducted in the last couple of years, examining a wide range of outcomes. From these studies, we learn that the effectiveness of feedback on performance is variable. While in some conditions feedback may be effective in enhancing performance related outcomes, in other conditions it may be ineffective or even decrease performance-related outcomes, e.g., [39,40]. Studies have found that effective feedback is specific, supportive and facilitative [41,42]. Specific feedback provides information about particular responses or behaviours. Supportive feedback encourages the recipient of feedback to take action or change behaviour and feedback that is facilitative offers guidance and cues on how to proceed [42].

This study adheres to these standards. Feedback offered in this study is *specific*, as the feedback is personalized and linked to the answers that individuals have given to the questions. It is *supportive*, in that it provides encouraging suggestions based on physicians’ qualities. Moreover, it includes both information about current performance, as well as specific suggestions about how to proceed and possible actions that physicians can take in order to stay able and willing to continue to work. Further, the report *is facilitative* as it offers guidance on how physicians could engage in job crafting by offering suggestions and ideas. This feedback facilitates reflection. This personalized guidance is likely to increase confidence. In turn, enhanced confidence is expected to positively affect motivational and behavioural outcomes [43], such as the ability and willingness to continue working and job-crafting behaviour.

The intervention that is offered in this study, using personalized feedback, fits the professional status of the participants well. Physicians are part of well-established professional fields that are largely self-regulatory [44,45]. Professionals are used to taking responsibility for their own learning process [46]. Furthermore, professionals’ large degree of autonomy [47] allows them to translate feedback into action.

Another reason why we expect that feedback in this study will enhance physicians’ employability and job-crafting behaviour is that these are topics where physicians do not usually reflect on or receive support as they describe their work environment as highly individualistic with poor mentorship and little support [48]. Physicians, like other professionals, have followed professional education. Therefore, they have neither been taught how to invest in their employability nor how to engage in job-crafting behaviour. In this sense, feedback on these topics is new to this group and may therefore be effective in improving employability scores and stimulating job crafting behaviour.

Based on the reasoning above, we expect that:

**Hypothesis** **1.** *At Time 2, physicians in the intervention group will report higher levels of physical ability to continue to work (a), mental ability to continue to work (b) and willingness to continue to work (c), compared to Time 1 as well as to a control group.*

**Hypothesis** **2.** *At Time 2, physicians in the intervention group will report higher levels of job crafting towards strengths (a), job crafting towards interests (b), job crafting to decrease hindering job demands (c) and job crafting to increase social job resources (d), compared to Time 1 as well as to a control group.*

## 2. Materials and Methods

### 2.1. Procedure and Participants

Physicians from two Dutch hospitals in a large city, i.e., one academic and one general hospital, participated in this study. A convenience sampling technique was used to recruit participants in the period from March until May 2019. We asked all departments within the two hospitals if we could communicate about the study and invite physicians to participate. Several departments agreed and let us hold a promotional presentation and distribute invitations through internal mailing list. In order to be included in the study, participants were required to work as a medical specialist in one of the two Dutch hospitals in which this study was conducted. Assuming a two-tailed significance level of 5% and a power level of 95%, power analysis indicated that 120 participants were needed to detect effect sizes of 0.25 and over. This effect size was based on the findings of research on full-scale job crafting interventions [20,21]. With a final sample size of 165 physicians, using a parallel design (ratio 50:50), we therefore had enough power to detect possible effects.

Physicians in the intervention group (*n* = 81) were predominantly female 64% (*n* = 51), on average 45.1 years old (SD = 8.01), contracted for 39.7 h per week (SD = 6.97), had been a physician for 10.5 years (SD = 7.85) and had been working in the organisation for 8.3 years (SD = 7.42). A total of 74% of the intervention group (*n* = 60) worked in an academic hospital. The composition of the control group (*n* = 84) was similar, with 65% females (*n* = 51), an average age of 45.0 years (SD = 8.20), contracted for 40.5 h per week (SD = 6.86), a mean functional tenure of 11.9 years (SD = 8.79), and a mean organisational tenure of 10.0 years (SD = 8.04). A total of 76% of the control group (*n* = 64) worked in an academic hospital. There were no significant differences between the intervention and control group on these demographics (all *p*’s > 0.17). 

### 2.2. Intervention

This study uses an experimental between-subject pretest-posttest design with random assignment to the waitlist control or intervention group. Apart from the assignment to the intervention or control group, all participants were treated similarly across the study process. Physicians were assigned to a group using the randomizer function in Microsoft Excel. Physicians were blind to the group that they were assigned to. Hospital type was the only criterion that was used to divide physicians in the control or intervention groups. Blocked randomization was used with hospital as a blocking factor. Blocked randomization is important given the expectation that physicians from both hospitals would differ in elements that might affect our dependent variables. For instance, all physicians in the academic hospital are employed by the hospital, while this applies to only a part of the physicians in the general hospital. In the general hospital, many physicians work as self-employed in independent establishments, running partnerships within hospitals, meaning that they do not report to a manager. Moreover, the HR department of the hospital does not develop HRM policies for this group. Additionally, the work of physicians in the academic hospital is usually more specialised than physicians working in the general hospital. Blocked randomization controls for possible variability caused by these differences by increasing the probability of equally dividing physicians from one hospital to the control or intervention group.

**Figure 1:**
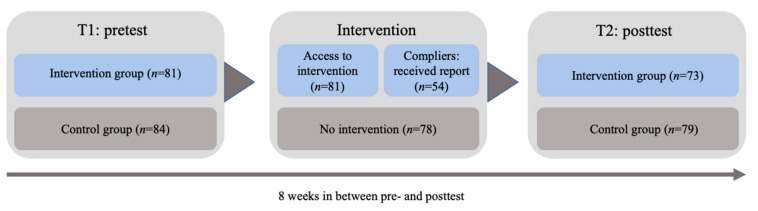
Intervention procedure.

Figure 1 shows the procedure of this intervention study. Participants received an e-mail inviting them to complete the pretest (T1) in May 2019. The survey started with a cover letter, stating the purpose of the study and assuring that responses would be kept confidential and that participation was voluntary. Participants provided informed consent by clicking on the “next” button. Both participants in the waitlist control group and the intervention group received this pretest. This pretest was separated from the additional questions that physicians in the intervention group received in order to obtain a personalized feedback report. After completing the pretest, physicians in the intervention group received a new link to the questions on which the personalized feedback report is based. This instrument was developed and tested in a previous cross-sectional study in the hospital context [49]. These questions asked about work experiences, well-being, and support of others in their work environment based on previously validated scales (e.g., see [49,50], which provide details on the content of these questions).

After physicians in the intervention group completed these questions (which took around 15 min), a personalized feedback report was automatically generated and presented. This personal feedback report consisted of approximately eight pages and provided a visual overview of a participant’s scores on (1) their actual work situation, showing their perception of their current ability and willingness to work, (2) the future work situation, showing their perception of internal and external career opportunities, (3) personal resources and (4) job demands and resources, including an indication of whether these scores were low, average or high. Additionally, a personalized set of examples of different sorts of job crafting behaviours was given. One example of how physicians could engage in job crafting to decrease hindering job demands is included by posing the following question: “You could ask experienced colleagues how they handle workload. Can you learn something from them?” An example of job crafting to increase social job resources is asking for social support or feedback [51]. This was for instance included in the report in the following advice: “You can discuss your struggles with a mentor who might fulfil the role of a discussion partner”. Examples of job crafting towards strengths and interests [22] were also included, such as: “If you struggle keeping up with certain work tasks, it could be helpful to search for new tasks that fit your strengths and interests”.

Additionally, ideas on how to increase employability were given. Possible causes of low scores were reported, and physicians were encouraged to reflect on these possible causes and take action. For example: “Social support, autonomy and task variety are resources that can be helpful to prevent exhaustion. You could think about changing these factors to increase your overall well-being to be able to continue working”. This shows how job crafting and employability were integrated in the personalized feedback received by physicians in the intervention group. Physicians reported that they spent on average five to thirty minutes on reading the report.

Eight weeks after the pretest (T1), physicians in the control and intervention groups received a link to the posttest (T2), in July 2019. For ethical reasons, physicians in the waitlist control group also received access to the personalized feedback report. They received this after the study was finished, so that it could not interfere with the study. The possibility of contamination between the control and intervention groups is reduced, as participants worked in different departments of the hospitals. Data were collected anonymously, with a code identifying each participant, so the data collected at the two measurement moments could be matched.

Figure 1 shows that not all physicians in the intervention group participated in the experiment as intended. All physicians in the intervention group had access to the intervention and were asked to complete questions to receive a personalized feedback report. Only 66.7% (*n* = 54) complied with these instructions and completed the questions and received a report. Several tests were conducted to examine whether these compliers differed on any of the study variables from participants in the intervention group that did not comply with the instructions. Multivariate analysis of variance indicated that there were no significant differences between these participants in terms of age, *F* (1,71) = 0.18, *p* = 0.95, hours worked according to contract, *F* (1,76) = 0.15, *p* = 0.22, functional tenure, *F* (1,77) = 0.19, *p* = 0.87, and organisational tenure, *F* (1,74) = 0.90, *p* = 0.69. Separate T-tests confirmed these findings. For the dichotomous variables gender and type of employment contract, we conducted chi-square tests, again showing that there were no significant differences between groups (all *p*s > 0.23). Therefore, an intention to treat analysis (ITT analysis) was conducted; that is, all randomized individuals were included in the analysis, regardless of whether they adhered to the study protocol [52]. In a non-study setting, when a program would be provided to employees, some employees are also likely to deviate from the predetermined plan. An ITT analysis therefore provides a good indication of what would happen in daily practice when such an intervention would be offered.

The intervention was developed in a previous study for hospital employees other than physicians [49]. For the present study, the intervention was refined to increase the fit with the needs and work context of physicians. The pre- and posttest, and the personalized feedback report were pilot-tested among five physicians. They were interviewed about the content, wording and style of addressing physicians in the report. If needed, the content of the feedback report and the item wordings were adapted. The largest changes that were made included adding the role of peers in offering support, besides managerial support. Adding the role of peers is important as physicians are often strongly committed to their profession [53]. Further, the advisory text was carefully reframed in terms of suggestions or encouragements instead of providing a top-down advice. This is believed to better fit the strong autonomy that physicians have.

### 2.3. Measures

The main study variables, ability and willingness to continue to work and job crafting, were measured at both T1 and T2. The control variables were measured at T1.

**Employability.** Physicians’ ability to continue working until retirement age (which is set at 67 years in the Netherlands) was measured with two items: “I am [physically (item 1)/mentally (item 2)] able to continue to work until the age of 67 in my current profession”. Willingness was measured with one item: “I am willing to continue to work until the age of 67 in my current profession” [6]. Answers were given on a 5-point Likert scale (1 = totally disagree, 5 = totally agree). Following previous research [54,55], the items asked physicians directly about their perceptions. Focusing on self-perceptions is relevant since people tend to act upon their perception rather than upon an objective reality [56,57].

**Job crafting.** Perceptions of job crafting behaviour are measured using two scales. The scale of Kooij et al. [22] focusses on job crafting directed to the person. This scale is used to measure job crafting towards strengths (4 items, e.g., “I organise my work in such a way that it matches with my strengths”; α_T1_ = 0.85; α_T2_ = 0.83), and job crafting towards interests (5 items, e.g., “I actively look for tasks that match my own interests”; α_T1_ = 0.86; α_T2_ = 0.84). The scale of Tims et al. [36] emphasizes job crafting regarding work aspects. This scale measures job crafting to increase social job resources (4 items, e.g., “I ask others for feedback on my job performance”; α_T1_ = 0.53; α_T2_ = 0.74) and job crafting to decrease hindering job demands (6 items, e.g., “I organise my work in such a way to make sure that I do not have to concentrate for too long a period at once”; α_T1_ = 0.68; α_T2_ = 0.72). We made some small changes to these scales prior to data collection, to make the questions applicable to physicians. A confirmatory factor analysis (CFA) was conducted to examine the factor structure and discriminant validity of the different scales of job crafting. We accepted the four-factor model, which distinguishes four different aspects of job crafting. Answers were given on a 5-point Likert scale (1 = never, 5 = very often).

**Control variables.** We included the following control variables: age, gender, type of employment contract, hours worked according to the contract, functional and organisational tenure.

### 2.4. Statistical Analysis

The study hypotheses were tested with two-way repeated measures multivariate analysis of variance (RM MANOVA) with Time (T1 versus T2) as a within-subject factor, and Group (control versus intervention group) and Type (type of job crafting) as between-subject factors. Subsequently, repeated measures ANOVAs were conducted (RM ANOVA) to examine the effects within the control and intervention groups. Table 1 presents the means, standard deviations, and correlations of the non-dichotomous study variables.

## 3. Results

### 3.1. Hypotheses Testing: Intervention and Employability

In the following, hypothesis 1 regarding physicians’ ability and willingness to continue work is tested. RM MANOVAs revealed a significant Time × Group interaction effect for both the perceived physical and mental ability to continue to work. For the physical ability to work, this interaction effect is slightly stronger (*F* (1,135) = 16.05, *p* < 0.001, partial *η*^2^ = 0.106) than for the mental ability to continue to work (*F* (1,130) = 4.57, *p* < 0.05, partial *η*^2^ = 0.034). No significant interaction effect was found for the willingness to continue to work.

**Figure 2:**
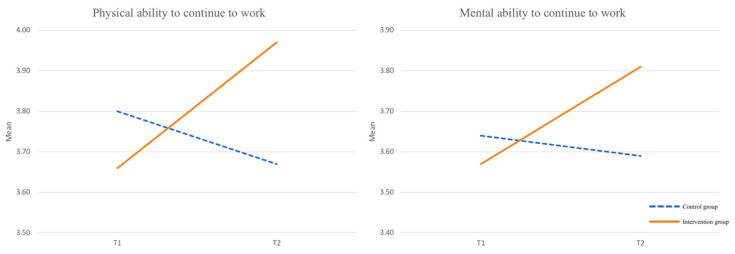
Results of RM ANOVAs for ability to continue to work.

We proceeded separately for the control and intervention group. RM ANOVA indicated a significant increase for the perceived physical ability to continue to work (*F* (1,66) = 15.287, *p* < 0.01) and for the perceived mental ability to continue to work (*F* (1,62) = 7.524, *p* < 0.01) within the intervention group from Time 1 to Time 2 (see Table 2 and Figure 2), compared to a non-significant decrease in the control group. This supports H1a and H1b. No significant across-time changes were found for the willingness to continue to work (*F* (1,61) = 2.033, *p* = 0.16) within the intervention group, rejecting H1c.

### 3.2. Hypotheses Testing: Intervention and Job Crafting

Below, hypothesis 2 regarding job crafting behaviours is tested. The results of RM MANOVAs are shown in Table 3. The outcomes revealed significant main effects for Time (*F* (1,138) = 9.87, *p* < 0.01) and Type (*F* (1,136) = 184.04, *p* < 0.01), as well as a significant Time × Type interaction effect (*F* (1,136) = 7.65, *p* < 0.01). The interaction effect between Time and Group was not significant (*F* (1,138) = 1.67, *p* = 0.20). This means that job-crafting behaviour increases significantly from Time 1 to Time 2 for physicians in both the control and intervention group. These effects differ across types of job crafting. The means presented in Table 1 show that both job crafting to decrease hindering job demands and job crafting towards strengths increased over time, regardless of group assignment. Job crafting to increase social job resources remained more or less stable, while job crafting towards interests increased slightly in the intervention group compared to a small decrease in the control group. Thus, although the participants overall reported higher levels of most types of job crafting behaviour across time, this increase could not be ascribed to the intervention (H2a-d not supported).

## 4. Discussion

This intervention study examined the effects of offering a personalized feedback report based on physicians’ perceptions of their employability and job-crafting behaviour. The results of a waitlist control group, receiving no intervention during the study, were compared to an intervention group receiving access to a personalized feedback report showing scores on their employability and suggestions for possible adjustments they could make in their work design.

### 4.1. Personalized Feedback Report and Employability

To examine the active ingredients of the intervention that caused the increase in physicians’ ability to continue to work, an additional analysis was performed, comparing the group of non-compliers with the compliers. A Time × Group × Type ANOVA revealed that the effect of Time did not vary across compliers vs. non-compliers. This, together with the finding that physicians in the intervention group reported higher levels of their physical and mental ability to continue to work compared to physicians in the control group, suggests that *offering* (rather than *reading*) the personalized feedback report already had a noticeable effect on the intervention outcomes.

Increased self-efficacy or self-confidence might explain the positive relationship between the personalized feedback report and physicians’ perceived ability to continue to work. Self-efficacy refers to the extent to which people feel confident about their abilities in specific task domains [58,59]. Physicians in the intervention group might have felt more confident about their abilities to continue to work, after having read the personalized feedback report. Future studies could examine empirically whether the relationship between an intervention, such as the one tested in this study, and perceived ability to continue to work is moderated by self-efficacy or self-confidence.

Another explanation for the increase in physicians’ perceptions on their ability to continue to work could be related to a learning process. Effective feedback has been shown to increase self-directed learning [60,61]. Self-directed learning is a process in which individuals take initiative in choosing and implementing appropriate learning strategies and evaluating learning outcomes [62]. In this study, physicians in the intervention group who received feedback might have learned about what actions they can take in order to stay employable in the future. This may in turn have resulted in more positive perceptions towards their ability to continue to work in their profession. Self-directed learning has been shown to be especially effective in advanced learners [63], such as physicians who are highly educated professionals. Future studies could examine the reasons for the increase in physicians’ ability to continue working further and see whether self-directed learning plays a role here.

An explanation for not finding a similar effect for the willingness to continue to work could be related to the fact that the intervention is mainly focused on work factors. Previous studies found that the willingness to continue to work, contrary to the ability to continue to work, is less influenced by work-related factors and mainly driven by non-work-related factors such as social support from a partner or financial reasons [54,55]. Future studies could examine this further, for instance by including additional advice and suggestions on non-work-related factors in the personalized feedback report.

### 4.2. Personalized Feedback Report and Job Crafting

This study shows that changes in job crafting varied for the different elements of job crafting. The non-significant outcomes for the relationship between the intervention and job crafting behaviour partly contrasts the findings of other job crafting intervention studies. A meta-analysis showed that job crafting interventions are in general effective in increasing job crafting behaviour with small effect sizes [15]. A possible explanation as to why we could not draw similar conclusions may be the intervention type. It is plausible that the outcomes of a high-investment training intervention with multiple sessions differ from those of a low-investment intervention. The source of feedback has been shown to affect outcome measures [60]. Physicians might be more motivated to engage in job crafting if the feedback comes from a person in their daily environment, rather than an unknown digital source. Future studies could examine if an intervention like the one examined in this study is effective in increasing job crafting behaviour if feedback is given in a different way, for instance by incorporating peer feedback in the personalized report.

In this study, job-crafting behaviour may not have been stimulated by the feedback in the personalized report, but by the questions that physicians in both the control and intervention group received in the pre- and posttest. This might explain the increase in perceptions of job crafting behaviour for physicians in both the control and intervention group. Comments of several physicians at the end of the posttest, saying that the questions in the pretest had triggered them to engage in job-crafting behaviour, support this idea. This is further supported by the non-significant findings of follow-up tests, showing that perceptions of job crafting increased for physicians in the intervention group, regardless of whether they complied with the study protocol. In sum, this suggests that merely asking respondents about their job-crafting behaviour may already function as an effective intervention.

### 4.3. Limitations and Future Research

This study has several limitations. First, a complication of randomized controlled intervention studies is the problem of noncompliance [64]. In this study, one-third of the participants did not adhere to the study protocol, meaning that they did not complete the questions to receive a personalized feedback report, despite receiving a reminder. These findings could well mirror what would happen in practice when this intervention would be offered in a non-study setting. Recent views on ITT analysis have argued that a better application of the ITT approach is possible if complete outcome data are available for all randomized subjects [64], i.e., even participants who did not comply with the treatment should be encouraged to complete the posttest. We have adhered to this advice. Many of the participants completed the posttest, regardless of compliance with the intervention protocol. The inclusion of all participants in the analyses may have diminished the possibility of finding effects. As the ITT analysis has presumably led to a conservative estimation of these effects, we can conclude that these effects of this intervention might well have been stronger if all participants would have complied with the study protocol.

Second, job crafting and employability were measured using self-reports, sometimes with a small number of items. Examining perceptions is common when measuring employability and job crafting, e.g., [6,56], and has been argued to be relevant, since people tend to act upon their perceptions [56,57]. Further, perceptions of employability by asking for employees’ ability and willingness to continue to work are good predictors of objective measures of similar concepts, such as early retirement in older workers [65]. From a practical point of view, this study relied on short scales, in line with our aim of developing an intervention that should not take much effort and time to complete. Methodologically, the disadvantage of this approach is that the reliability of some measures in this study could not be determined and will be lower than that of multi-item measures. However, this implies that the associations between these measures and other concepts were estimated conservatively: it is likely that the effects reported in this study would have been stronger if more sophisticated measures had been used. Yet, it would certainly be of interest for future research to examine whether similar conclusions can be drawn when using multi-item scales and/or objective indicators for employability.

Third, the physicians could have two different types of employment contract, that is, they could either be employed by the hospital or work as a self-employed worker in partnerships with other specialists. Due to a lack of statistical power, we could not examine whether there were any statistical differences in the effectiveness of the personalized feedback report for physicians with various types of contracts. Future studies may further investigate this, as two recent studies have shown that contract type may affect employees’ mental health and physical health [66] and the relationship between job crafting and employability [67].

Finally, this study included only one follow-up measurement, eight weeks after the pretest. The ideal time between a pre- and posttest in order to measure changes in perceptions on the ability and willingness to continue to work and job crafting is unclear. A time frame in existing studies varies from one or two weeks e.g., [3] to eight weeks e.g., [21]. In a theory on evaluating training programs, Kirkpatrick and Kirkpatrick argue that an individual’s response to training can be split into four levels: reaction, learning, behaviour, and results [68]. In our study, we were especially interested in the third level, namely the extent to which participants change their behaviour as a result of the intervention. According to Kirkpatrick and Kirkpatrick, the levels are sequential, and more time is needed to detect changes in behaviour, compared to observing changes in reaction and learning [68]. Since we are interested in changes in perceived behaviour, and this is only a low-investment intervention, a period of eight weeks between the pre- and posttest seemed appropriate. This study confirms this as the outcomes show that changes already took place within this period. Future studies could include an additional measurement moment to examine if some variables might have needed more time to be detected.

### 4.4. Study Implications

First, this study enriches the literature on employability. The randomized controlled intervention study is, to our knowledge, the first experimental study that examines how this can be enhanced. The findings of this study provide insights into the factors that may increase physicians’ employability.

Second, the importance of employees who proactively manage their work in order to stay employable has been widely acknowledged in the international debate on careers, e.g., [69,70]. This study contributes to the current body of knowledge on job crafting interventions by testing a low-investment intervention, which is less expensive and time-consuming to implement than the active interventions tested in previous studies. Despite the low level of effort put in, it seems that asking questions about job-crafting behaviour may be effective in enhancing job crafting perceptions. More studies into the effects of low-investment interventions are needed to examine other types of job crafting interventions besides the job crafting training intervention.

Third, this study is valuable for practice, especially since this field experiment is tested in a natural setting [71] and since it is a low-investment intervention that is relatively cheap and easy to implement and does not take much effort to complete. Digital feedback is especially attractive to use in highly demanding work setting [72]. This tool can be used for various health care professionals. It could, for instance, be used for doctors as a part of their performance system. Awareness of the importance of attention for physicians’ mental health, wellbeing and their ability to remain working in their profession has increased worldwide [73]. Demanding working conditions in many countries challenge the employability of this group. They are increasingly expected to fulfil various roles and proactively manage their work, as is for instance reflected in the various roles expressed in the CanMEDS Physician Competency Framework [74]. This tool can help physicians to do this by collecting ideas on how to invest in their employability, which they might include in their personal development plan that they have to develop. This tool might additionally provide input for the annual appraisal conversation between managers and physicians.

## 5. Conclusions

This intervention study shows promising results for enhancing physicians’ perceptions of their ability to continue to work. Offering physicians a personal feedback report increased perceptions of their mental and physical ability to continue to work. Moreover, this study contributes to the literature by examining another approach of a job crafting intervention. This low-investment intervention increased physicians’ perceptions of their job crafting behaviour significantly over time, but the personalized feedback report did not explain this change. Rather it seems that the survey that includes questions on job crafting behaviour enhanced physicians’ perceptions of their job-crafting behaviour. This study provides insights about how to encourage physicians’ employability and job-crafting behaviour through this low-investment intervention. 

## Figures and Tables

**Table 1:** Means, standard deviations and Pearson correlations of the main study variables.

	Control Group		Intervention Group													
	M	SD	M	SD	1	2	3	4	5	6	7	8	9	10	11	12
1. JC-decreasing hindering job demands—at T1	1.63	0.46	1.59	0.41	1	0.51 **	0.10	−0.08	0.26 *	0.07	0.39 **	0.27 *	0.01	−0.02	0.05	−0.08
2. JC-decreasing hindering job demands—at T2	1.90	0.71	1.93	0.88	0.30 **	1	−0.03	−0.06	−0.00	0.03	0.11	0.11	0.08	0.05	0.04	−0.16
3. JC-increasing social job resources—at T1	2.73	0.60	2.82	0.49	0.25 *	0.12	1	0.49 **	0.24 *	0.16	0.23 *	0.14	−0.15	0.04	−0.00	−0.04
4. JC-increasing social job resources—at T2	2.71	0.67	2.75	0.51	0.31 **	0.14	0.69 **	1	−0.03	0.19	0.02	0.21	−0.12	0.19	−0.09	0.03
5. JC-towards strengths at T1	3.19	0.73	3.17	0.81	0.15	0.18	0.23 *	0.23 *	1	0.74 **	0.63 **	0.46 **	0.27 *	0.16	0.24 *	0.29 *
6. JC-towards strengths at T2	3.29	0.61	3.29	0.84	0.07	0.19	0.20	0.28 *	0.67 **	1	0.48 **	0.65 **	0.29 *	0.29 *	0.16	0.22
7. JC-towards interests at T1	3.08	0.75	2.98	0.73	0.39 **	0.17	0.41 **	0.33 **	0.66 **	0.42 **	1	0.64 **	0.29 *	0.33 **	0.31 **	0.39 **
8. JC-towards interests at T2	3.01	0.71	3.18	0.75	0.30 *	0.11	0.35 **	0.41 **	0.56 **	0.63 **	0.67 **	1	0.40 **	0.41 **	0.36 **	0.37 **
9. Ability to continue to work at T1	3.71	0.78	3.57	0.88	0.02	−0.04	0.26 *	0.13	0.12	0.15	0.23 *	0.26 *	1	0.76 **	0.47 **	0.44 **
10. Ability to continue to work at T2	3.61	0.77	3.90	0.85	−0.04	0.01	0.31 **	0.16	0.04	0.11	0.16	0.21	0.70 **	1	0.54 **	0.55 **
11. Willingness to continue to work at T1	2.97	1.18	3.23	1.12	0.21	0.24	0.35 **	0.19	0.27 *	0.21	0.25 *	0.15	0.43 **	0.40 **	1	0.80 **
12. Willingness to continue to work at T2	2.92	1.12	3.29	1.17	0.15	0.14	0.22	0.04	0.14	0.21	0.22	0.18	0.50 **	0.52 **	0.76 **	1

Note, results for the control group (*n* = 84) are shown under the diagonal. Results for the intervention group (*n* = 81) are shown above the diagonal. * Correlation is significant at the 0.05 level (2-tailed), ** correlation is significant at the 0.01 level (2-tailed).

**Table 2:** Results of RM ANOVAs for ability and willingness to continue to work.

	Intervention Group (*n* = 81) Means		RM ANOVA		Control Group (*n* = 84) Means		RM ANOVA	
	T1	T2	*F*-Values	*ƞ* ^2^	T1	T2	*F*-Values	*ƞ* ^2^
Physical ability to continue to work	3.66	3.97	*F* (1,66) = 15.287, *p* < 0.01	0.188	3.80	3.67	*F* (1,69) = 2.868, *p* = 0.10	x
Mental ability to continue to work	3.57	3.81	*F* (1,62) = 7.524, *p* < 0.01	0.108	3.64	3.59	*F* (1,68) = 0.198, *p* = 0.66	x
Willingness to continue to work	3.19	3.32	*F* (1,61) = 2.033, *p* = 0.16	x	2.99	2.88	*F* (1,66) = 1.090, *p* = 0.30	x

**Table 3:** Results of RM MANOVAs for Time × Group × Type of job-crafting behaviour.

	Job-Crafting Behaviour	
	*F*-Values	*p*
Time	*F* (1,138) = 9.87	0.002 **
Time × Group	*F* (1,138) = 1.67	0.198
Type	*F* (1,136) = 184.04	0.000 **
Type × Group	*F* (1,136) = 0.23	0.877
Time × Type	*F* (1,136) = 7.65	0.000 **
Time × Type × Group	*F* (1,136) = 1.33	0.267

** significant at the 0.01 level (2-tailed).

## Data Availability

The datasets analysed during the current study are available from the corresponding author on reasonable request.
